# Kinesitherapeutic Behavior on the Operative Treatment of Impingement Syndrome

**Published:** 2019-08

**Authors:** Maria VAKRILOVA BECHEVA, Angelina KIRKOVA-BOGDANOVA

**Affiliations:** 1. Department of Nursing, Faculty of Public Health, Medical University of Plovdiv, Plovdiv, Bulgaria; 2. Department of Medical Informatics, Biostatistics and E-Learning, Faculty of Public Health, Medical University of Plovdiv, Plovdiv, Bulgaria

## Dear Editor-in-Chief

The shoulder joint is the most mobile one in the human body, allowing for a variety of movements. Because of a distortion in the biomechanics or problems in the shoulder joint, in certain movements, it is possible for the soft tissues to be pressed between the shoulder bone and the acromial growth of the blade and the condition is termed as impingement syndrome.

Typical symptoms of the impingement syndrome include difficulty in placing the hand behind the back, pain when elevating the arm and placing it behind the head and weakness of the muscles in the shoulder belt area. The so-called “painful arc”, characteristic of impingement syndrome, is associated with pain in part of the movement when the arm is lifted upward (1). The Neer, Hawkins and Yocum tests are used to precise the topography of the lesioned tendons in the rotator cuff. The Jobe test objectifies the suppression of m.suprascapularis tendon, the Patte’s test objectifies the lesion of the m. infraspinatus tendon, a lift-off test proves the engagement of the tendon to m. subscapularis and a palm-up test objectifies the lesion of the m. biceps brachii-caput longum tendon (2).

The aim of the presented article was to familiarize the audience with the applied means in the various stages of the kinesiotherapy applied in the post-operative treatment of impingement syndrome.

The indications for surgical treatment are:

➢ Closed sub-biochemical space, leading to the squeezing of tissues beneath the acromion.
➢ Stage II of impingement syndrome (Neer’s classification) with the presence of pronounced fibrosis or calcifications in the subacromial space (3).
➢ Minimum rotator cuff tear, calcifications in the muscles of the rotator cuff.
➢ Unsuccessfully conservative rehabilitation treatment for 3 to 6 months.

Postoperative kinesitherapy takes place in three stages: maximum protective stage, moderate and minimal protective stage. Immobilization after arthroscopic treatment is carried out with an arm cast, when taken up to the body and an inwardly turned arm and collapsed to 90° elbow. In the maximally protective stage of kinesitherapy, the arm cast is removed and exercises are performed the next day after surgery. For glenohumeral joint support, we use passive or active assisted exercises for armpit flexion in the scapula plane, in a painless volume of movement (usually 90° to 120°) from the first day after surgery. Godman pendulum exercises are applied without weights. Auto-assisted exercises with a stick are recommended. If muscle disinsection and reinsertion is performed during the operation, active movement of the respective muscle is carried out minimally for at least two weeks (up to 6 weeks) to protect the healing tissue. To restore the control and muscle strength of the shoulder complex the treatment should begin with submaximal multi-round isometric exercises for the muscles of the scapula, the rotator cuff, and the rest of the muscles of the glenohumeral joint (4). To maintain the strength of the muscles stabilizer of the scapula the treatment should apply rhythmic stabilization exercises. Postural correction exercises are included as early as possible (1).

In the moderate and minimal protective stage, kinesitherapy progresses rapidly. The treatment is performed in a controlled active traffic volume. By the sixth postoperative week the patient can restore the full active volume of movement of the shoulder complex. At the beginning of the moderate protective stage, free active exercises are applied and exercises against resistance are gradually included. If dynamic exercises against resistance are painful, multivariate isometric exercises against resistance are applied (4). At sixth postoperative week, open-closed and closed kinetic resistance exercises are included. If movement-limiting structures exist, active inhibitory techniques - relaxation, stretching, and self-restraining exercises are included. Stretching of the rear joint capsule is applied if the horizontal adduction and flexion of the armpit are limited. The kinesitherapeutic program progresses towards training the strength and endurance of muscles in functional motor models by increasing the time, speed and intensity of performance (5).

The application of kinesitherapy in the different phases promotes the progressive recovery of passive and active subnormal joint amplitudes realized according to the physiological scheme and allowing functional independence of the patient. Patient’s expectations need to be taken into account. If there are no complications, the moderate protection phase may end by the end of the fourth month. Applying kinesitherapy helps to improve the muscular strength of the shoulder complex, including force-related activities. By means of kinesitherapy, the patient readapts to specific professional and sports activities (6). Applying kinesitherapy helps restore the control and muscle strength of the shoulder complex and improve the function of the healthy parts.
